# BMI category, recorded diagnosis composition, and occupational medicine certificate validity: the POLOCARIA study, 2016–2025

**DOI:** 10.3389/fpubh.2026.1920503

**Published:** 2026-10-21

**Authors:** Marcin Poboży, Anna Rulkiewicz, Wojciech Lisik, Zoran Stojčev, Ewa Filipiak-Rokicka, Iwona Pilchowska, Justyna Domienik-Karłowicz

**Affiliations:** 1 NZOZ Cichowski Poboży Healthcare Facility, Maciejowice, Poland; 2 LUX MED Group, Warsaw, Poland; 3 Department of General, Oncological, Metabolic and Thoracic Surgery, CSK MON WIM-PIB, Warsaw, Poland; 4 Second Chair and Department of General, Vascular and Oncological Surgery, Faculty of Medicine and Dentistry, Medical University of Warsaw (WUM), Warsaw, Poland; 5 Warsaw University of Medical Sciences, Warsaw, Poland; 6 Department of Internal Medicine and Cardiology, Medical University of Warsaw, Warsaw, Poland

**Keywords:** blood pressure, body mass index, hyperglycaemia, obesity, occupational health certificate, occupational medicine, real-world data

## Abstract

**Background:**

Occupational medicine examinations offer an opportunity to identify cardiometabolic risk among professionally active adults. This study examined associations between BMI, recorded diagnosis composition, circulatory parameters, and occupational medicine certificate validity.

**Methods:**

This retrospective observational study analyzed 988,822 patient-year records from 620,882 patients attending a large private healthcare network in Poland during 2016–2025. Associations were examined using chi-square tests, analysis of variance, Spearman correlations, and linear regression with standard errors clustered by patient.

**Results:**

Mean systolic blood pressure increased from 121.65 mmHg at normal weight to 141.01 mmHg in Class III obesity, while mean certificate validity decreased from 34.81 to 27.80 months. Diabetes/hyperglycaemia diagnoses represented 8.2% of selected diagnoses at normal weight and 20.8% in Class III obesity. BMI was negatively correlated with certificate validity (ρ = −0.172) and positively correlated with systolic blood pressure (ρ = 0.401). In the adjusted model, age category (β = −0.209), systolic blood pressure (β = −0.148), and BMI (β = −0.053) were associated with shorter certificate validity.

**Conclusions:**

Higher BMI was associated with higher blood pressure, greater representation of selected cardiometabolic diagnoses, and shorter certificate validity. Occupational examinations may support cardiometabolic risk recognition, but diagnosis percentages are not prevalence estimates and certificate validity is not a measure of work ability or a causal consequence of BMI.

## Graphical Abstract

**Figure:**
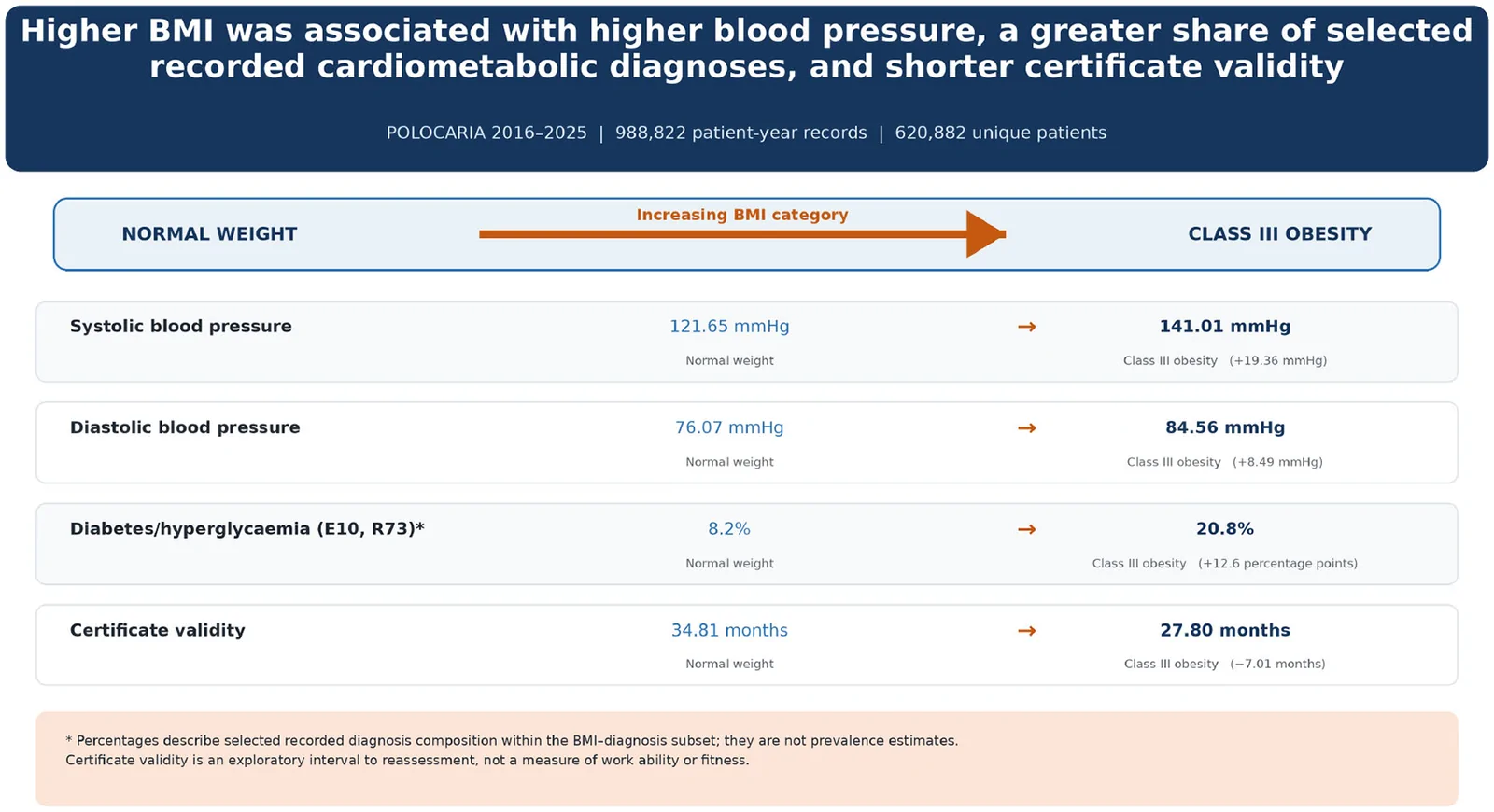
Summary of the principal associations observed in the POLOCARIA 2016–2025 dataset.

## 1 Introduction

Overweight and obesity are major public-health challenges and important determinants of non-communicable disease. Their prevalence has increased substantially worldwide, including across Europe, creating particular challenges for prevention in working-age populations (1–3).

Body mass index (BMI) remains the most widely available measure of excess weight in population and clinical datasets. Although it does not describe body composition or fat distribution, higher BMI is consistently associated with hypertension, impaired glucose metabolism, dyslipidaemia, and cardiovascular disease (4–9).

The consequences of obesity also extend to occupational health. Previous studies have linked higher BMI with poorer work ability, sickness absence, productivity loss, and occupational injury, supporting the value of weight and cardiometabolic risk assessment in workplace health programmes (10–19).

Occupational medicine examinations provide repeated opportunities to identify elevated BMI, blood pressure, and other cardiometabolic risk indicators among professionally active adults. Routinely collected health records can therefore complement conventional surveillance, provided that their clinical and administrative context is recognized (20, 21). The validity period of an occupational medicine certificate is one such context-dependent measure: it indicates the interval to reassessment rather than work ability or fitness for a particular job.

The earlier POL-O-CARIA 2016–2020 study described obesity and severe obesity and their relationship with cardiovascular comorbidities among professionally active adults (22). The present study extends observation through 2025 and examines a distinct question: how BMI relates to recorded diagnosis composition, circulatory parameters, and certificate validity.

This study aimed to examine associations between BMI category, selected recorded diagnoses, blood pressure, heart rate, and occupational medicine certificate validity in a large patient-year dataset from Poland.

## 2 Materials and methods

### 2.1 Study design and population

This retrospective observational study analyzed routinely collected occupational-health records from a large private healthcare network in Poland between January 2016 and December 2025. The analytical unit was the patient-year, with one record per patient in each calendar year. Patients with records in more than one year were therefore represented more than once. The study included 988,822 patient-year records from 620,882 unique patients.

The analyzed variables were continuous BMI, BMI category, age category, sex, province of residence, smoking status, systolic and diastolic blood pressure, heart rate, certificate validity (months), and selected ICD-10 diagnoses. Variable-specific denominators are presented in Table 1. Analyses used available observations, without imputation.

**Table 1:** Analytical sample and availability of variables in the 2016–2025 patient-year dataset.

Dataset component	*n*	% of patient-year records
Patient-year records	988,822	100.0
Unique patients	620,882	–
Patients represented in ≥2 calendar years	221,985	35.8% of unique patients
Continuous BMI	933,929	94.5
BMI category	909,565	92.0
Sex	517,386	52.3
Selected diagnostic category	517,386	52.3
BMI category and diagnostic category	473,335	47.9
Certificate validity	984,321	99.5
Systolic blood pressure	959,157	97.0
Diastolic blood pressure	925,854	93.6
Heart rate	834,678	84.4

### 2.2 BMI and diagnostic categories

BMI was analyzed continuously and categorized in accordance with the standard World Health Organization (WHO) classification for adults: underweight (< 18.5 kg/m^2^), normal weight (18.5 to < 25.0 kg/m^2^), overweight (25.0 to < 30.0 kg/m^2^), Class I obesity (30.0 to < 35.0 kg/m^2^), Class II obesity (35.0 to < 40.0 kg/m^2^), and Class III obesity (≥40.0 kg/m^2^) (1). Selected diagnoses were grouped into predefined ICD-10 categories (Supplementary Table S1). One diagnosis was recorded per patient-year; therefore, category percentages reflect the composition of recorded diagnoses rather than the true prevalence of disease.

### 2.3 Certificate validity

Certificate validity was defined as the duration, in months, of the occupational medicine certificate. It was treated as an exploratory clinical-administrative interval to reassessment, not as a measure of work ability, job-specific fitness, disease severity, or prognosis.

### 2.4 Statistical analysis

Categorical variables were summarized as frequencies and percentages, while continuous variables were described using standard descriptive statistics. The associations between categorical variables were evaluated with chi-square tests and Bonferroni-adjusted comparisons of column proportions. Differences across BMI categories were analyzed through one-way analysis of variance, complemented by Scheffé *post hoc* comparisons and eta-squared (η^2^) effect size estimates. Spearman's correlation coefficients were utilized to characterize relationships between BMI, certificate validity, and circulatory parameters.

Linear regression models examined certificate validity concerning age category, systolic blood pressure, and continuous BMI. Standard errors and 95% confidence intervals were computed with clustering by patient identifier. Statistical analyses were performed using IBM SPSS Statistics version 25 and Python. Due to the extensive sample size, interpretation focused on effect sizes as well as the magnitude and direction of the associations.

## 3 Results

### 3.1 Study population

The dataset consisted of 988,822 patient-year records derived from 620,882 distinct patients (see Table 1). A total of 221,985 patients (35.8%) were documented across at least two calendar years. Among the 517,386 records with gender information, 53.4% pertained to women and 46.6% to men. The Body Mass Index (BMI) category was available for 909,565 records, and 473,335 records included both BMI category and a specified diagnosis.

### 3.2 BMI and recorded diagnosis composition

The composition of recorded diagnoses varied across BMI categories (χ^2^ (65, *N* = 473,335) = 42,995.75, *p* < 0.001, *V* = 0.135). The corresponding association for detailed ICD-10 codes was χ^2^ (1180, *N* = 473,335) = 57,928.39 (*p* < 0.001, *V* = 0.156).

Diabetes/hyperglycaemia diagnoses (E10 and R73) represented 8.2% of selected diagnoses at normal weight and increased progressively to 20.8% in Class III obesity. Cardiovascular/thromboembolic diagnoses increased from 2.7% at normal weight to 5.9% with overweight and peaked at 6.8% in Class I obesity. Gout (M10) increased from 0.7% at normal weight to 6.1% in Classes II and III obesity (Table 2; Figure 1).

**Table 2:** Distribution of selected recorded diagnosis categories by BMI category (% within BMI category; *n* = 473,335).

Diagnosis category	Normal	Overweight	Class I	Class II	Class III	Total
Diabetes/hyperglycaemia (E10, R73)	8.2	15.0	18.7	20.4	20.8	12.9
Selected dermatological (L40, L73)	6.3	6.2	5.0	4.8	4.5	6.0
Ovarian dysfunction (E28)	4.2	1.9	2.0	2.4	3.0	3.0
Gastro-esophageal/hepatobiliary	19.0	21.4	19.9	17.8	15.3	19.8
Reproductive/menstrual disorders	15.8	5.9	4.7	5.2	6.6	10.1
Cardiovascular/thromboembolic	2.7	5.9	6.8	6.3	6.0	4.6
Chronic kidney disease (N18)	0.4	0.5	0.5	0.4	0.3	0.4
Cerebrovascular disorders	0.4	0.4	0.4	0.3	0.3	0.4
Abnormal blood chemistry (R79)	3.5	2.8	2.4	2.2	1.8	3.0
Depressive/anxiety disorders	18.0	12.2	9.7	9.0	10.0	14.3
Asthma/COPD (J44, J45)	12.7	12.6	11.3	11.3	11.8	12.3
Gout (M10)	0.7	3.1	5.3	6.1	6.1	2.6
Osteoarthritis (M15–M19)	6.0	9.5	10.7	11.0	9.8	8.2
Sleep disorders	2.3	2.5	2.6	2.8	3.7	2.5

The underweight category is omitted from the displayed table for clarity. Percentages describe recorded diagnosis composition and are not prevalence estimates.

**Figure 1:**
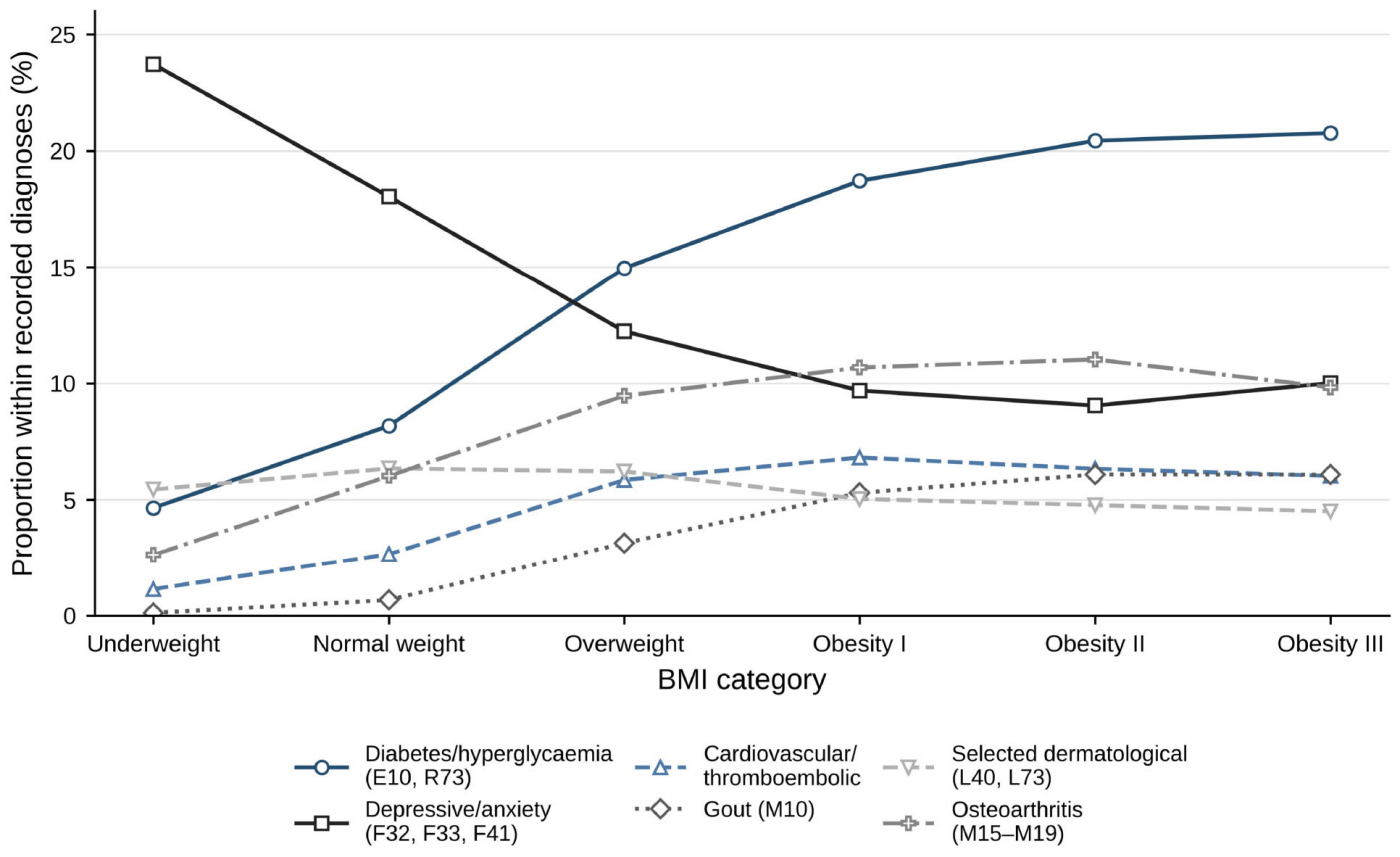
Proportions of selected recorded diagnosis categories within each BMI category.

Depressive and anxiety disorders (F32, F33, and F41) accounted for 18.0% of the diagnosed cases among individuals with normal weight, 12.2% among those with overweight, and approximately 9%−10% within the obesity categories. These figures represent the distribution of selected recorded diagnoses, rather than the prevalence of mental health disorders within the BMI groups.

### 3.3 BMI, circulatory parameters, and certificate validity

Systolic blood pressure demonstrated variation across BMI categories (F(5, 885,952) = 27,199.44, *p* < 0.001, η^2^ = 0.133). Similarly, diastolic blood pressure (F (5, 854,858) = 17,598.44, *p* < 0.001, η^2^ = 0.093), heart rate (F (5, 778,033) = 1,383.38, *p* < 0.001, η^2^ = 0.009), and certificate validity (F (5, 905,509) = 4,539.94, *p* < 0.001, η^2^ = 0.024) also exhibited significant differences, as presented in Table 3.

**Table 3:** One-way analysis of variance for clinical variables by BMI category.

Variable	*n*	df	*F*	*p*	η^2^
Systolic blood pressure	885,958	5; 885,952	27,199.44	< 0.001	0.133
Diastolic blood pressure	854,864	5; 854,858	17,598.44	< 0.001	0.093
Heart rate	778,039	5; 778,033	1,383.38	< 0.001	0.009
Certificate validity (months)	905,515	5; 905,509	4,539.94	< 0.001	0.024

η^2^ is the effect-size measure; *p* values are unadjusted for repeated records.

The mean systolic blood pressure elevated from 121.65 mmHg in individuals with normal weight to 130.10 mmHg among those with overweight, further rising to 134.69 mmHg in Class I obesity, 137.89 mmHg in Class II obesity, and reaching 141.01 mmHg in Class III obesity. Additionally, the mean diastolic blood pressure increased from 76.07 mmHg to 84.56 mmHg (refer to Figure 2).

**Figure 2:**
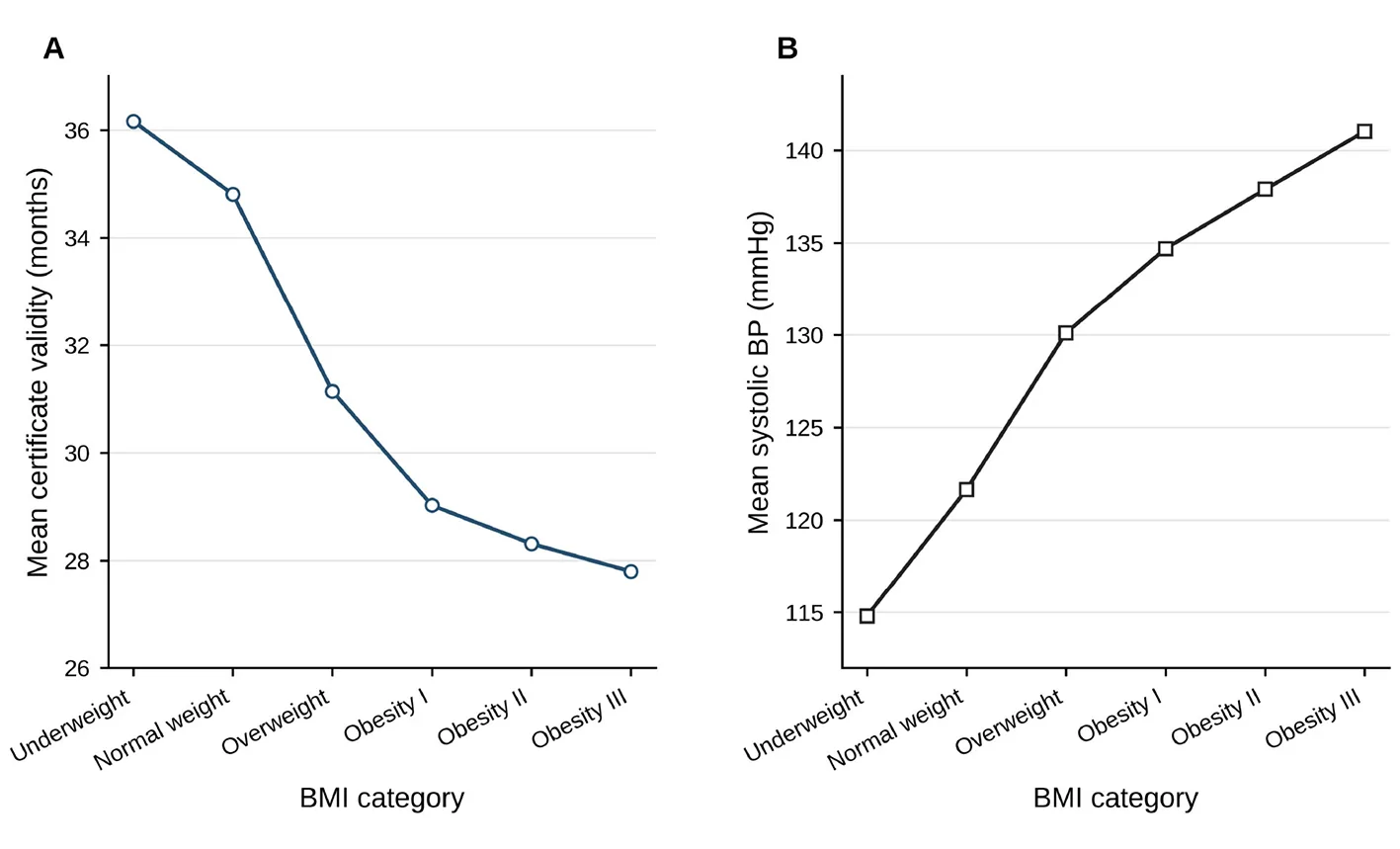
Mean **(A)** occupational medicine certificate validity period and **(B)** systolic blood pressure by BMI category.

The mean certificate validity diminished from 34.81 months for individuals of normal weight to 31.14 months among those who are overweight, 29.02 months in Class I obesity, 28.31 months in Class II obesity, and 27.80 months in Class III obesity (see Figure 2). The variance between normal weight and Class III obesity was 7.01 months.

### 3.4 Patterns over time

Between 2016 and 2025, the proportion of records classified as normal weight declined from 46.0 to 39.1%. Conversely, the proportion of overweight records rose from 33.9 to 35.3%, and the combined prevalence of Classes I–III obesity increased from 17.5 to 23.2% (see Figure 3). These statistics delineate the annual distribution of patient-year records within the participating healthcare network.

**Figure 3:**
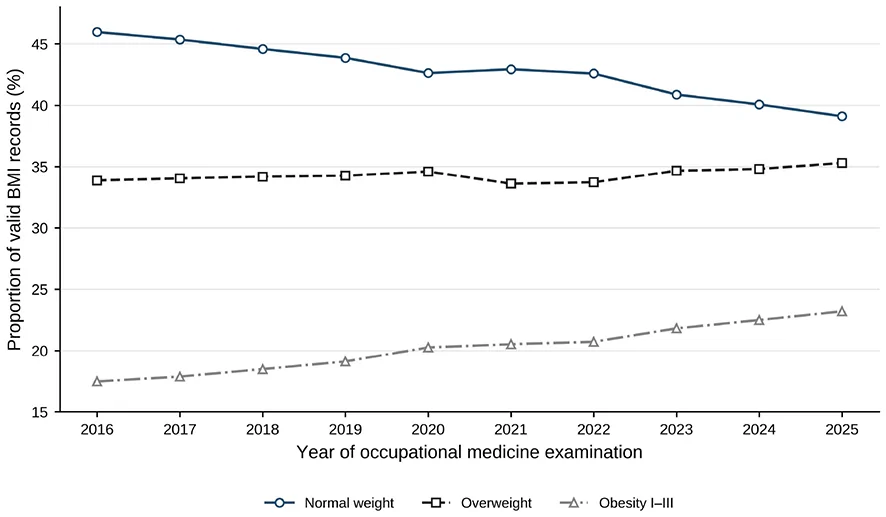
Annual proportions of normal weight, overweight, and class I–III obesity among patient-year records with a valid BMI category, 2016–2025.

The average certificate validity increased in 2021, reached its peak in 2022, and subsequently declined through 2025. Among records containing sex information, the mean certificate validity was longer for females than for males in each year of the study (Figure 4).

**Figure 4:**
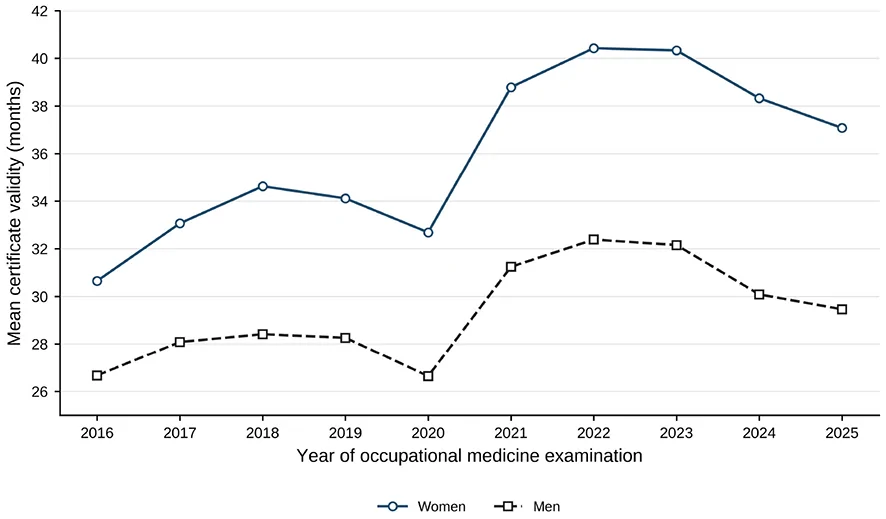
Mean occupational medicine certificate validity by year and sex among records with available sex and certificate data, 2016–2025.

### 3.5 Correlation and regression analyses

Body Mass Index (BMI) was inversely correlated with certificate validity (ρ = −0.172, *p* < 0.001; *n* = 929,798) and positively correlated with systolic blood pressure (ρ = 0.401, *p* < 0.001; *n* = 909,960), diastolic blood pressure (ρ = 0.347, *p* < 0.001; *n* = 878,224), and heart rate (ρ = 0.087, *p* < 0.001; *n* = 800,074) (see Table 4; Figure 5). Diagnosis-specific analyses demonstrated similar directional associations, with detailed estimates provided in Tables 4, 5.

**Figure 5:**
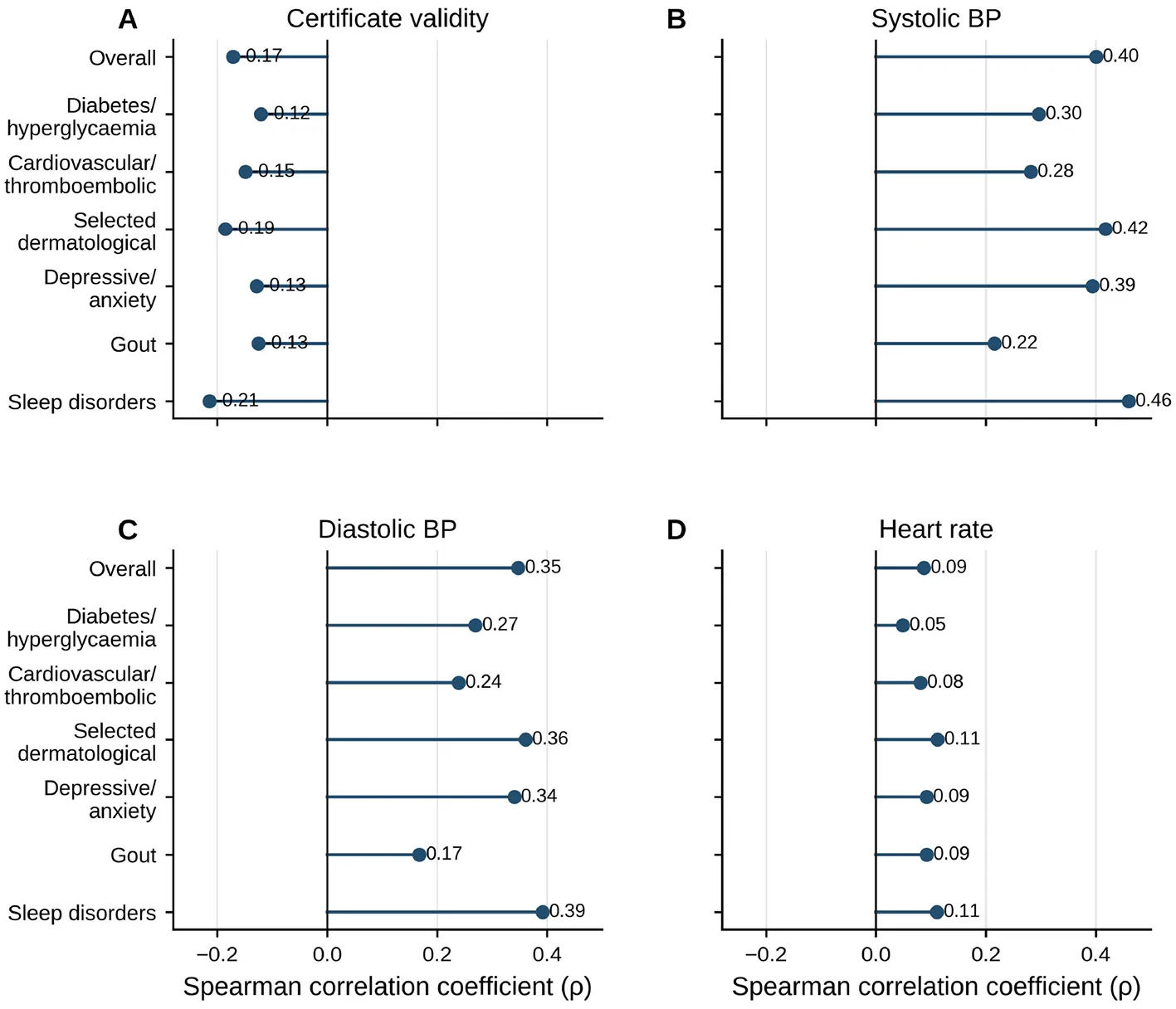
Spearman correlations of BMI with **(A)** certificate validity, **(B)** systolic blood pressure, **(C)** diastolic blood pressure, and **(D)** heart rate in the overall sample and selected diagnostic categories.

**Table 4:** Spearman correlations of BMI with certificate validity and circulatory parameters.

Category	Variable	ρ	*p*	*N*
Overall	Certificate validity	−0.172	< 0.001	929,798
Diabetes/hyperglycaemia (E10, R73)	Certificate validity	−0.121	< 0.001	62,882
Selected dermatological	Certificate validity	−0.185	< 0.001	28,841
Cardiovascular/thromboembolic	Certificate validity	−0.149	< 0.001	22,350
Depressive/anxiety disorders	Certificate validity	−0.129	< 0.001	69,119
Gout	Certificate validity	−0.125	< 0.001	12,704
Overall	Systolic blood pressure	0.401	< 0.001	909,960
Diabetes/hyperglycaemia (E10, R73)	Systolic blood pressure	0.297	< 0.001	62,012
Selected dermatological	Systolic blood pressure	0.418	< 0.001	28,127
Cardiovascular/thromboembolic	Systolic blood pressure	0.282	< 0.001	22,007
Depressive/anxiety disorders	Systolic blood pressure	0.394	< 0.001	67,341
Gout	Systolic blood pressure	0.216	< 0.001	12,449
Overall	Diastolic blood pressure	0.347	< 0.001	878,224
Diabetes/hyperglycaemia (E10, R73)	Diastolic blood pressure	0.270	< 0.001	57,422
Selected dermatological	Diastolic blood pressure	0.361	< 0.001	27,227
Cardiovascular/thromboembolic	Diastolic blood pressure	0.239	< 0.001	21,126
Depressive/anxiety disorders	Diastolic blood pressure	0.341	< 0.001	66,076
Gout	Diastolic blood pressure	0.168	< 0.001	11,390
Overall	Heart rate	0.087	< 0.001	800,074
Diabetes/hyperglycaemia (E10, R73)	Heart rate	0.049	< 0.001	55,345
Selected dermatological	Heart rate	0.112	< 0.001	24,294
Cardiovascular/thromboembolic	Heart rate	0.081	< 0.001	18,947
Depressive/anxiety disorders	Heart rate	0.092	< 0.001	59,911
Gout	Heart rate	0.093	< 0.001	10,978

Correlation estimates are descriptive.

**Table 5:** Associations of BMI with selected diagnostic domains and certificate validity.

Clinical domain	Normal	Overweight	Class III	ρ validity	ρ SBP	*R* ^2^	β BMI	β age	β SBP
Cardiovascular/thromboembolic	2.7	5.9	6.0	−0.149	0.282	0.136	−0.083	−0.314	−0.087
Diabetes/hyperglycaemia	8.2	15.0	20.8	−0.121	0.297	0.103	−0.020	−0.205	−0.193
Gout	0.7	3.1	6.1	−0.125	0.216	0.115	−0.086	−0.281	−0.118
Depressive/anxiety	18.0	12.2	10.0	−0.129	0.394	0.047	−0.063	−0.153	−0.084
Sleep disorders	2.3	2.5	3.7	−0.214	0.460	0.106	−0.120	−0.227	−0.092

Proportions describe selected recorded diagnosis composition. Standardized β estimates derive from models adjusted for age category and systolic blood pressure, with standard errors clustered by patient.

In the overall regression model, age category, systolic blood pressure, and BMI explained 9.7% of the variance in certificate validity. Age exhibited the largest standardized coefficient (β = −0.209, 95% CI −0.211 to −0.206), followed by systolic blood pressure (β = −0.148, 95% CI −0.151 to −0.146) and BMI (β = −0.053, 95% CI −0.055 to −0.050); (Tables 6, 7; Figure 6). BMI maintained a negative association with certificate validity within the selected categories of cardiovascular/thromboembolic, diabetes/hyperglycaemia, depressive/anxiety, dermatological, and gout conditions (Table 7).

**Table 6:** Goodness-of-fit of linear regression models for certificate validity.

Diagnostic category	*R* ^2^	Adjusted *R*^2^	SE
Overall	0.097	0.097	15.04
Cardiovascular/thromboembolic	0.136	0.135	13.53
Diabetes/hyperglycaemia (E10, R73)	0.103	0.103	13.91
Depressive/anxiety disorders	0.047	0.047	15.78
Selected dermatological	0.089	0.089	14.86
Gout	0.115	0.115	14.36

**Figure 6:**
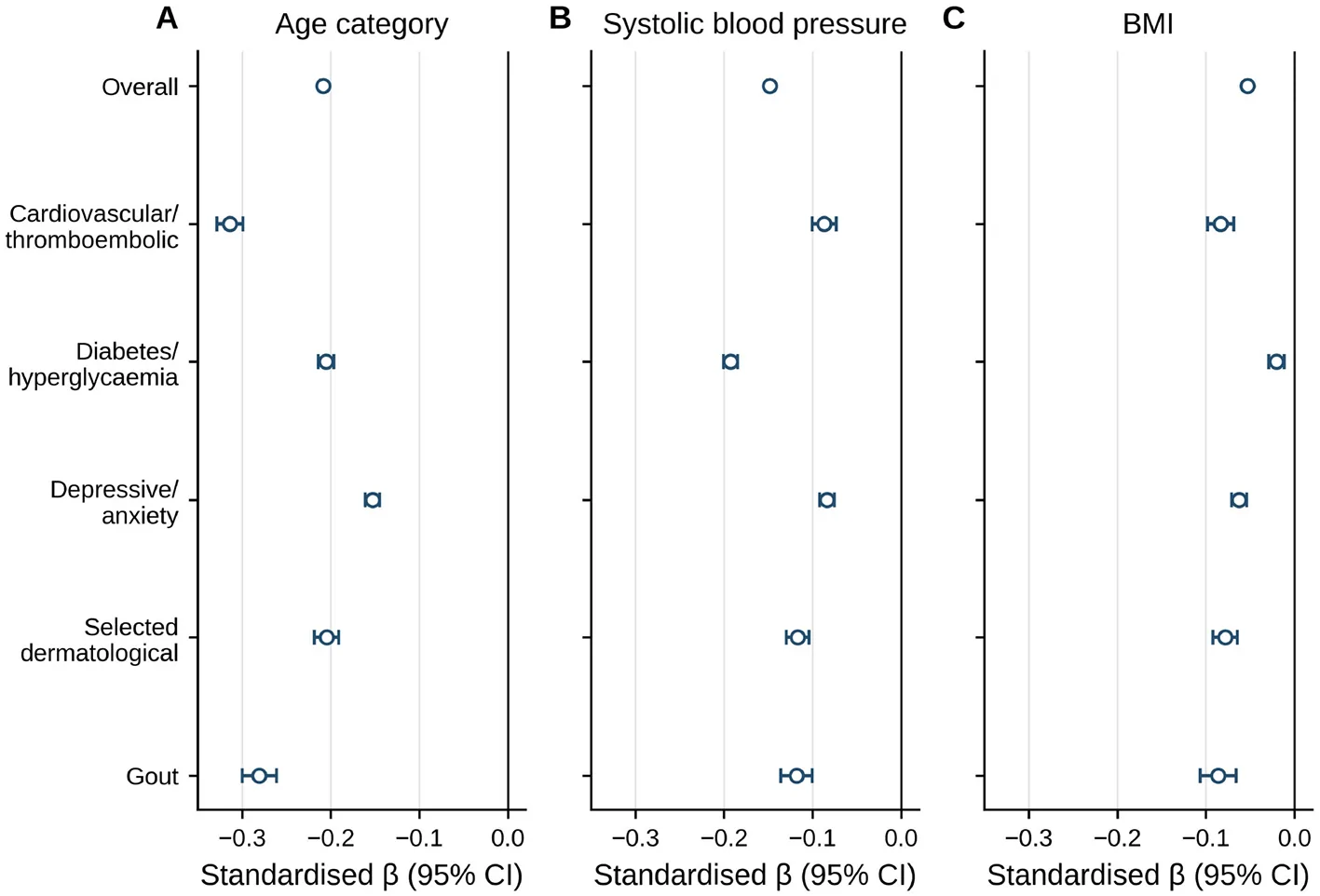
Forest plots of standardised β coefficients with 95% confidence intervals for associations of **(A)** age category, **(B)** systolic blood pressure, and **(C)** BMI with certificate validity in the overall sample and selected diagnostic categories.

**Table 7:** Selected linear regression coefficients for certificate validity.

Category	Covariate	B	Robust SE	β	95% CI for β	*p*
Overall	Age category	−2.684	0.016	−0.209	−0.211 to −0.206	< 0.001
Overall	Systolic blood pressure	−0.143	0.001	−0.148	−0.151 to −0.146	< 0.001
Overall	BMI	−0.164	0.004	−0.053	−0.055 to −0.050	< 0.001
Cardiovascular/thromboembolic	Age category	−4.178	0.100	−0.314	−0.328 to −0.299	< 0.001
Cardiovascular/thromboembolic	Systolic blood pressure	−0.078	0.006	−0.087	−0.100 to −0.073	< 0.001
Cardiovascular/thromboembolic	BMI	−0.245	0.022	−0.083	−0.098 to −0.068	< 0.001
Diabetes/hyperglycaemia	Age category	−2.554	0.055	−0.205	−0.214 to −0.197	< 0.001
Diabetes/hyperglycaemia	Systolic blood pressure	−0.156	0.003	−0.193	−0.200 to −0.185	< 0.001
Diabetes/hyperglycaemia	BMI	−0.056	0.012	−0.020	−0.029 to −0.012	< 0.001
Depressive/anxiety disorders	Age category	−2.345	0.065	−0.153	−0.161 to −0.145	< 0.001
Depressive/anxiety disorders	Systolic blood pressure	−0.094	0.005	−0.084	−0.092 to −0.076	< 0.001
Depressive/anxiety disorders	BMI	−0.201	0.014	−0.063	−0.071 to −0.054	< 0.001
Selected dermatological	Age category	−2.971	0.102	−0.205	−0.218 to −0.191	< 0.001
Selected dermatological	Systolic blood pressure	−0.115	0.006	−0.117	−0.130 to −0.104	< 0.001
Selected dermatological	BMI	−0.245	0.022	−0.078	−0.092 to −0.065	< 0.001
Gout	Age category	−4.028	0.142	−0.281	−0.300 to −0.261	< 0.001
Gout	Systolic blood pressure	−0.109	0.008	−0.118	−0.136 to −0.100	< 0.001
Gout	BMI	−0.263	0.032	−0.086	−0.107 to −0.066	< 0.001

Standard errors, confidence intervals, and *p*-values are robust to clustering by patient identifier.

## 4 Discussion

This extensive occupational health study identified a consistent gradient across Body Mass Index (BMI) categories. Elevated BMI levels were associated with increased systolic and diastolic blood pressure, a higher prevalence of specific cardiometabolic diagnoses, and a reduced duration of certificate validity. The disparities between normal weight and Class III obesity were significant, with differences of 19.36 mmHg in systolic blood pressure, 8.49 mmHg in diastolic blood pressure, and a reduction of 7.01 months in certificate validity.

The blood-pressure gradient aligns with established evidence linking adiposity with hypertension and cardiovascular disease (4, 6, 9, 23–25). It additionally underscores the significance of occupational examinations as opportunities to identify elevated blood pressure and to initiate suitable follow-up procedures. Nonetheless, the results should be regarded as associations rather than definitive evidence that BMI directly causes the observed circulatory differences.

The diagnostic findings provide a supplementary perspective. The rising proportion of E10/R73 diagnoses across various BMI categories is consistent with the well-established association between excess adiposity and impaired glucose metabolism (26–29). The concomitant increase in gout is also clinically plausible, given its metabolic correlations (30). However, as only one diagnosis was recorded per patient-year, these percentages reflect the composition of coding rather than the prevalence of individual diseases or multimorbidity.

The smaller proportion of depressive and anxiety diagnoses observed at higher BMI should not be construed as indicative of a lower prevalence of mental disorders among individuals with obesity. Depression, anxiety, and obesity frequently coexist, and their interrelationships are intricate and bidirectional (31–33). The pattern identified may more accurately reflect the increased prevalence of somatic diagnoses within the selected diagnostic field.

The certificate's validity period was inversely correlated with BMI both before and after adjustment for age category and systolic blood pressure, although the standardized BMI coefficient remained minimal. This interval reflects a combination of clinical and administrative determinations made by an occupational physician and may vary depending on the examination context, job requirements, occupational exposures, and regulatory standards. Consequently, it should be regarded as the re-evaluation timing, rather than a direct measure of work capability or fitness.

The present analysis expands upon the previously issued POL-O-CARIA report by extending the observation timeframe to the year 2025. Additionally, it incorporates data on recorded diagnoses, circulatory measurements, and certificate validity. While it does not provide estimates of individual BMI trajectories or national prevalence rates, it effectively illustrates the potential of occupational health records to uncover clinically consistent patterns within a large working population.

The findings support a preventive strategy focused on verifying elevated blood pressure, evaluating glycaemic and cardiovascular risk when clinically justified, implementing evidence-based weight management, and promoting non-discriminatory workplace health initiatives (16, 17, 23, 24, 27, 29, 34). Body Mass Index (BMI) should be used as an initial risk indicator alongside other clinical data and should not be used in isolation to assess occupational fitness.

## 5 Strengths and limitations

The primary strengths of this investigation include its extensive sample size, decade-long coverage, objective anthropometric and circulatory assessments, and the incorporation of patient-clustered uncertainty estimates within the regression models. However, its retrospective observational design limits causal inference, and data obtained from a single private healthcare network may not be representative of all occupational health providers or the entire professionally active population of Poland.

The patient-year structure encompassed repeated observations for certain patients. Clustering was accounted for in the regression models; however, it was not considered in the chi-square, analysis of variance, or correlation analyses, and thus, these should be regarded solely as descriptive. The regression models were not adjusted for job characteristics, occupational exposures, examination type, smoking status, sex, or calendar year. Furthermore, selected diagnoses were documented for 52.3% of patient-years and reflected a single diagnosis rather than comprehensive morbidity data. These aspects may introduce selection and coding biases.

BMI does not account for body composition or fat distribution, and the validity of certificates is affected by clinical, occupational, and regulatory factors outside the scope of the models. Consequently, the findings should be regarded as population-level associations within the participating network rather than as individual prognostic or fitness evaluations (7, 8, 18).

## 6 Conclusions

A higher Body Mass Index (BMI) is associated with increased blood pressure, a greater number of specific cardiometabolic diagnoses, and shorter validity periods for occupational medicine certificates. As BMI rises from normal weight to Class III obesity, the average systolic blood pressure increases by 19.36 mmHg, while the average certificate validity decreases by 7.01 months. These results support using occupational exams to identify and refer individuals at risk for cardiometabolic conditions. However, it is crucial to understand that the recorded diagnosis percentages do not reflect actual disease prevalence, and the certificate validity duration should not be viewed as an indicator of work capacity or as directly caused by BMI.

## Data Availability

The data analysed in this study are not publicly available because access is subject to institutional permissions and applicable data-protection requirements. Requests to access the data should be directed to the corresponding author and will be considered subject to permission from the data controller and applicable legal and institutional requirements.

## Supplementary material

Supplementary Table S1 ICD-10 codes included in each diagnostic category and category-specific observation counts.
