# Supplementary material for: BMI category, recorded diagnosis composition, and occupational medicine certificate validity: the POLOCARIA study, 2016–2025
Source: Front Public Health. 2026 Oct 21;14:1920503. doi: 10.3389/fpubh.2026.1920503 (PMC13731241; doi:10.3389/fpubh.2026.1920503)
Supplement: Supplementary file 1 [file Table_1.docx]

**Supplementary Table S1**

**ICD-10 codes included in each selected diagnostic category, observation counts, and clinical rationale**

Counts refer to patient-year records from 2016–2025 for which both a BMI category and a selected diagnosis were recorded (N = 473,335). Categories describe the composition of selected recorded ICD-10 diagnoses and should not be interpreted as prevalence estimates.

| **Diagnostic category** | **Included ICD-10 codes** | **n** | **Clinical rationale** |
| --- | --- | --- | --- |
| Diabetes/hyperglycaemia | E10; E10.0–E10.9; R73; R73.0; R73.9 | 61,277 | Type 1 diabetes codes and abnormal glycaemia findings. This selected group does not include all diabetes diagnoses and must not be interpreted as overall diabetes prevalence. |
| Selected dermatological disorders | L40; L40.0–L40.5; L40.8–L40.9; L73; L73.0–L73.2; L73.8–L73.9 | 28,195 | Psoriasis and selected follicular disorders; kept separate because the extracted codes did not cover all dermatological disease. |
| Ovarian dysfunction | E28; E28.0–E28.3; E28.8–E28.9 | 14,141 | Ovarian dysfunction, androgen excess, and related endocrine-reproductive diagnoses. |
| Gastro-oesophageal/hepatobiliary disorders | K21; K21.0; K21.9; K76; K76.0–K76.1; K76.4–K76.9; K80; K80.0–K80.5; K80.8 | 93,583 | Gastro-oesophageal reflux, selected liver disease including fatty liver, and gallstone disease. |
| Reproductive/menstrual disorders | N46; N91; N91.0–N91.5; N92; N92.0–N92.6; N93; N93.0; N93.8–N93.9; N97; N97.0–N97.2; N97.4; N97.8–N97.9 | 48,040 | Male infertility, female infertility, and menstrual or uterine bleeding disorders. |
| Selected cardiovascular/thromboembolic disorders | I21; I21.0–I21.4; I21.9; I22; I25; I25.0–I25.3; I25.5–I25.6; I25.8–I25.9; I26; I26.0; I26.9; I48; I50; I50.0–I50.1; I50.9; I80; I80.0–I80.3; I80.8–I80.9; I82; I82.0–I82.3; I82.8–I82.9 | 21,889 | Myocardial infarction, ischaemic heart disease, pulmonary embolism, atrial fibrillation, heart failure, phlebitis, and venous thrombosis. |
| Chronic kidney disease | N18; N18.0; N18.8–N18.9 | 1,947 | Chronic kidney disease and kidney failure. |
| Cerebrovascular disorders | G45; G45.0–G45.4; G45.6; G45.8–G45.9; I63; I63.0–I63.6; I63.8–I63.9; I64 | 1,874 | Transient ischaemic attack, cerebral infarction, and unspecified stroke. |
| Abnormal blood chemistry findings | R79; R79.0; R79.8–R79.9 | 14,075 | Abnormal blood chemistry findings without a single specified disease entity. |
| Depressive and anxiety disorders | F32; F32.0–F32.3; F32.8–F32.9; F33; F33.0–F33.4; F33.8–F33.9; F41; F41.0–F41.3; F41.8–F41.9 | 67,478 | Depressive episodes, recurrent depressive disorder, and anxiety disorders. The category does not represent all mental health diagnoses. |
| Asthma and COPD | J44; J44.0–J44.1; J44.8–J44.9; J45; J45.0–J45.1; J45.8–J45.9 | 58,107 | Chronic obstructive pulmonary disease and asthma. |
| Gout | M10; M10.0–M10.4; M10.9 | 12,387 | Gout diagnoses only. |
| Osteoarthritis | M15; M15.0–M15.4; M15.8–M15.9; M16; M16.0–M16.7; M16.9; M17; M17.0–M17.5; M17.9; M18; M18.0–M18.5; M18.9; M19; M19.0–M19.2; M19.8–M19.9 | 38,719 | Polyosteoarthritis and selected hip, knee, thumb-base, and other osteoarthritis diagnoses. |
| Sleep disorders | G47; G47.0–G47.4; G47.8–G47.9 | 11,623 | Insomnia, hypersomnia, circadian-rhythm disorders, sleep apnoea, narcolepsy, and other selected sleep disorders. |
